# Bowel associated dermatosis – arthritis syndrome: a case report

**DOI:** 10.1186/1752-1947-1-81

**Published:** 2007-09-05

**Authors:** Dayavathi Ashok, Patrick Kiely

**Affiliations:** 1 Rheumatology, Buckinghamshire Hospitals NHS Trust, High Wycombe, UK; 2 Rheumatology, St George's Healthcare NHS Trust, London, UK

## Abstract

We report a rare case of Bowel Associated Dermatosis – Arthritis Syndrome in a young patient with complex Crohn's disease who presented with fever, arthritis, rash and worsening of diarrhea with abdominal pain, who promptly responded to a short course of steroids.

## Case Presentation

A 23 year old white male with a 4 year history of Crohn's disease presented with an acute two day history of malaise, fever, abdominal pain, vomiting and stomal diarrhoea. He complained of joint pains affecting the shoulders, elbows, wrists, metacarpophalangeals, knees and ankles. There was also a rash on the elbows, ankles and feet, which began as erythematous macules and evolved to vesico-pustular lesions followed by crusting. There was no history of sexual exposure or any intercurrent infection. He was on no regular medication, but had discontinued Pentasa 4 months earlier.

One month earlier he had undergone a laparotomy to excise a complex ileo-cutaneous fistula with blind tracts, and two weeks earlier a defunctioning ileostomy had been created in view of persistent abdominal pain. The cutaneous fistula had been present for a year, but was associated with a terminal ileal stricture and ileo-rectal fistula of at least 2 years duration. His bowel disease had been resistant to immunosuppressive drugs including azathioprine, corticosteroiods and three infusions of Infliximab a year earlier. There had been no extra-intestinal manifestations.

**Figure 1:**
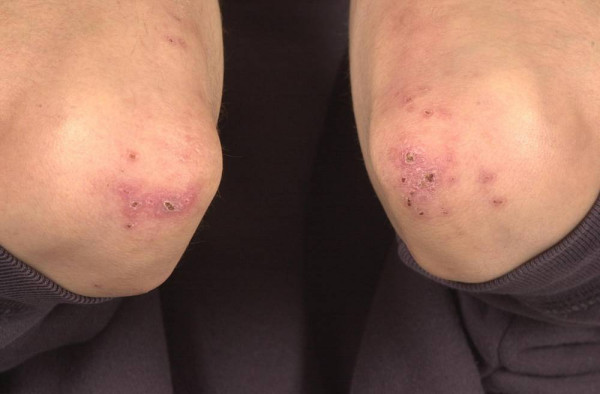
Pustules and crusts around the elbows.

**Figure 2:**
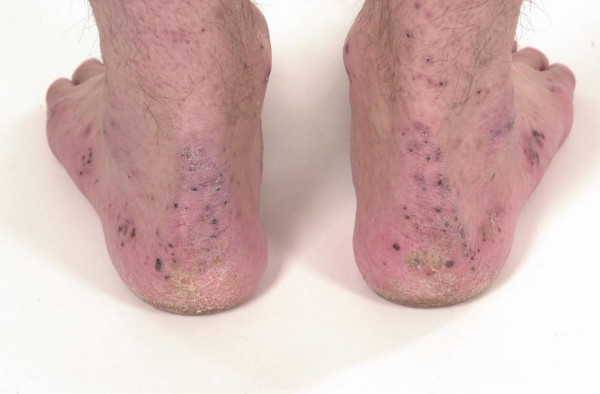
Crusts around the ankles and feet.

On admission to the hospital, he was thin, afebrile with a resting tachycardia of 125/minute. The rest of the cardio-respiratory examination was normal. The abdomen was minimally tender around the ileostomy without guarding or rebound tenderness. Examination of the skin revealed some pustules and crusts around the elbows, ankles and feet (Fig 1 and 2). The buttocks were spared. There were clinical signs of synovitis of the wrists, proximal interphalangeal and metacarpophalangeal joints, and also both ankles.

Results of the laboratory tests showed a haemoglobin of 13.1 gm/dl, white blood count 15.8 × 10^9^/L, platelets 585 × 10^9^/L, C- reactive protein 37.7 mg/L and erythrocyte sedimentation rate 69 mm/hr. Urea and electrolytes, complement, urine analysis and microscopy were normal. Rheumatoid factor, antinuclear and antineutrophil cytoplasmic antibodies, cryoglobulins and Hepatitis B and C serology were negative. Transthoracic echocardiogram showed no signs of endocarditis, and multiple blood cultures were sterile. A skin biopsy from the ankle revealed a perivascular lymphohistiocytic infiltrate with prominent neutrophils and associated fibrinoid necrosis of vessels consistent with 'leukocytoclastic' vasculitis.

He was commenced on 60 mg prednisolone per day leading to prompt and complete resolution of all features. The dose of prednisolone was rapidly tapered over the course of one month by the patient, faster than advised but without any recurrence over the following 3 years.

## Discussion

The "bowel bypass syndrome" is a well recognized complication of jejunoileal bypass surgery for obesity. This is characterized by an influenza-like illness in 10–20% of patients, with malaise, fever, non-erosive polyarthralgia or arthritis and the development of skin lesions [1,2]. It has been proposed that bacterial overgrowth in a blind loop of bowel results in the formation of immune complexes, which enter the circulation and produce the clinical features [3-5]. The characteristic skin lesions appear in crops as small erythematous macules (less than 1 cm diameter) progressing through an indurated urticarial stage to vesiculo-pustular lesions over a 24 – 48 hour period, healing without scarring over about 2 weeks. Lesions may recur every 4 – 6 weeks, and on the lower limbs erythema nodosa-like nodules may be present [2,6]. The distribution classically favours the upper trunk and extremities, and histology shows features of a neutrophilic dermatosis, similar to Sweet's syndrome, with leukocytoclasis but no fibrinoid necrosis [2,6]. Joint symptoms are described as episodic, migratory and polyarticular, with involvement of the fingers and accompanying tenosynovitis, but no long term damage or deformity [2].

A similar clinicopathological syndrome has been reported rarely in patients with other bowel conditions such as inflammatory bowel disease. In 1983, Jorizzo and associates [6] described four patients with an identical syndrome, who had not undergone jejunoileal bypass surgery; however, each patient had gastrointestinal disease. Subsequently this syndrome has also been described as a complication of inflammatory bowel disease in a further 10 cases in the literature [7-10]. The term 'bowel associated dermatosis-arthritis syndrome' has therefore been proposed to include patients without bowel bypass surgery for obesity. However, all of the published cases shared the risk factor of bowel stasis to promote bacterial overgrowth, with either disease or surgically induced blind loops.

Bowel associated dermatosis-arthritis syndrome should be distinguished from Henoch-Schonlein purpura (HSP) which, whilst typically a disease of childhood, can affect adults. The distinguishing features of HSP are the lack of association with pre-existing bowel disease, the presence of glomerulonephritis, and the immunoglobulin A (IgA) deposition in the skin, glomeruli and gastrointestinal mucosa [11]. The pathogenic mechanisms underlying HSP are poorly understood, and whilst they may include gut infection, other mucosal mechanisms such as drugs or hypersensitivity have been postulated to lead to abnormalities in IgA synthesis [12].

Dermatological manifestations of Crohn's disease include erythema nodosum, pyoderma gangrenosum and more rarely a cutaneous granulomatous vasculitis. The latter usually affects the distal lower limbs in association with asymmetric large joint arthritis, fever abdominal pain and diarrhoea [13]. This is distinguished from the bowel-associated Dermatosis-Arthritis syndrome in which skin histology has not been reported as showing granulomas and where the distribution of rash favours the upper limbs and joint disease includes small joints [14].

Other than steroids, various antibiotics including tetracycline, minocycline, sulphapyridine, erythromycin and metronidazole have been reported to suppress symptoms in the Bowel Associated Dermatosis-Arthritis Syndrome, although the response to these antibiotics has been inconsistent [2,15]. Restoration of normal bowel anatomy has also been curative though in many cases the syndrome is self limiting [2].

## Conclusion

We report a case of bowel associated dermatosis-arthritis syndrome in a patient with complicated Crohn's disease, a long standing ileo-rectal fistula and the recent creation of a defunctioning ileostomy. Our patient responded promptly to the administration of corticosteroids and maintained remission for 3 years, similar to previous reports in the literature. Although cutaneous leukocytoclastic vasculitis is a non specific manifestation of a wide variety of pathologic processes [16] the other features of this syndrome taken together (systemic upset, abdominal pain, and the distribution of arthritis and skin lesions) are diagnostic, and their recognition may prevent the need to undertake exhaustive investigations.

## Competing interests

The author(s) declare that they have no competing interests.

## Authors' contributions

Both the authors made equal contribution.

All the authors read and approved the final manuscript.
